# Lung MRI and impairment of diaphragmatic function in Pompe disease

**DOI:** 10.1186/s12890-015-0058-3

**Published:** 2015-05-06

**Authors:** Stephan CA Wens, Pierluigi Ciet, Adria Perez-Rovira, Karla Logie, Elizabeth Salamon, Piotr Wielopolski, Marleen de Bruijne, Michelle E Kruijshaar, Harm AWM Tiddens, Pieter A van Doorn, Ans T van der Ploeg

**Affiliations:** Department of Neurology, Erasmus MC, Rotterdam, The Netherlands; Centre for Lysosomal and Metabolic Diseases, Erasmus MC-Sophia, Rotterdam, The Netherlands; Department of Radiology, Erasmus MC, Rotterdam, The Netherlands; Department of Pediatrics, Respiratory Medicine and Allergology, Erasmus MC-Sophia, Rotterdam, The Netherlands; Department of Radiology, Beth Israel Deaconess Medical Center- Harvard Medical School, Boston, MA USA; Biomedical Imaging Group Rotterdam, Departments of Radiology and Medical Informatics, Erasmus MC, Rotterdam, The Netherlands; Department of Pediatric Pulmonology, Erasmus MC-Sophia, Rotterdam, The Netherlands; Department of Computer Science, University of Copenhagen, Copenhagen, Denmark; Department of Pediatrics, Division of Metabolic Diseases and Genetics, Erasmus MC-Sophia, Rotterdam, The Netherlands

**Keywords:** Pompe disease, Glycogen storage disease type II, Lysosomal storage disorder, MRI, Diaphragm, Pulmonary function, Spirometry

## Abstract

**Background:**

Pompe disease is a progressive metabolic myopathy. Involvement of respiratory muscles leads to progressive pulmonary dysfunction, particularly in supine position. Diaphragmatic weakness is considered to be the most important component. Standard spirometry is to some extent indicative but provides too little insight into diaphragmatic dynamics. We used lung MRI to study diaphragmatic and chest-wall movements in Pompe disease.

**Methods:**

In ten adult Pompe patients and six volunteers, we acquired two static spirometer-controlled MRI scans during maximum inspiration and expiration. Images were manually segmented. After normalization for lung size, changes in lung dimensions between inspiration and expiration were used for analysis; normalization was based on the cranial-caudal length ratio (representing vertical diaphragmatic displacement), and the anterior-posterior and left-right length ratios (representing chest-wall movements due to thoracic muscles).

**Results:**

We observed striking dysfunction of the diaphragm in Pompe patients; in some patients the diaphragm did not show any displacement. Patients had smaller cranial-caudal length ratios than volunteers (p < 0.001), indicating diaphragmatic weakness. This variable strongly correlated with forced vital capacity in supine position (r = 0.88) and postural drop (r = 0.89). While anterior-posterior length ratios also differed between patients and volunteers (p = 0.04), left-right length ratios did not (p = 0.1).

**Conclusions:**

MRI is an innovative tool to visualize diaphragmatic dynamics in Pompe patients and to study chest-walland diaphragmatic movements in more detail. Our data indicate that diaphragmatic displacement may be severely disturbed in patients with Pompe disease.

**Electronic supplementary material:**

The online version of this article (doi:10.1186/s12890-015-0058-3) contains supplementary material, which is available to authorized users.

## Background

Pompe disease (OMIM 232300: acid maltase deficiency or glycogen storage disease type II) is an inherited progressive metabolic myopathy caused by acid α-glucosidase deficiency due to mutations in the acid α-glucosidase (GAA) gene (OMIM 606800) [1,2]. Pulmonary dysfunction caused by progressive weakness of the respiratory muscles is a characteristic feature of the disease [1,3,4]. In patients with the classic infantile form cardiorespiratory failure leads to death within the first year of life [5,6]. In patients with late-onset or non-classic Pompe disease pulmonary dysfunction progresses more slowly. The first sign of respiratory involvement in these patients is decreased pulmonary function in supine position, eventually necessitating respiratory support during sleep. Patients in the end-stage of the disease require continuous respiratory support [7-9]. Weakness of the diaphragm–the main respiratory muscle–is considered to be the major cause of respiratory dysfunction in Pompe disease [3,10]. Although pulmonary function tests (PFTs) may be indicative of diaphragmatic weakness by showing a difference between forced vital capacity (FVC) in sitting and supine position–i.e. postural drop–or by a decreased mean inspiratory pressure (MIP), they provide too little insight in dynamics of the diaphragm [11]. More insight in the function of the diaphragm has become extra relevant since enzyme replacement therapy (ERT) has been available for Pompe disease. While several studies have shown that ERT has positive effects on skeletal muscle function by showing stabilization or improvement of muscle strength or the distance walked in six minutes, the effects on lung function especially in supine position seem to be less pronounced [7,12-16]. In an earlier study that compared the effects of ERT on pulmonary function in sitting and supine positions, we found that 15% of patients were therapy resistant when pulmonary function was measured in sitting position, and that 35% were therapy resistant when it was measured in supine position [13]. Recent MRI sequences and image analysis techniques make it possible to directly assess the individual contribution of respiratory muscles–including the diaphragm–during the breathing cycle [17-22].

The aim of the current study was to determine whether MRI could be used as an innovative tool to gain greater insight into the function of the diaphragm in Pompe disease, and to correlate these data with the results of PFTs.

## Methods

### Study population

All patients with Pompe disease in the Netherlands are referred to Erasmus MC University Medical Centre Rotterdam. For this cross-sectional pilot study we selected ten adult patients with various degrees of respiratory dysfunction. As controls we included six age- and gender-matched volunteers. Informed consent was obtained from all participants. The study protocol was approved by the Medical Ethical Committee at our hospital (Amendment 7 to protocol MEC-2007-103).

### PFT

An MRI-compatible spirometer was used to standardize lung volumes and breathing movements during the MRI (MasterSceen Pneumo spirometer, CareFusion, Houten, the Netherlands). Before MRI, FVC and forced expiratory volume in one second (FEV_1_) were measured according to ATS/ERS standards [23,24]. Spirometry parameters are expressed in percentages predicted. Postural drop (ΔFVC) was calculated as (FVC_sitting_-FVC_supine_)/FVC_sitting *_100%. An ΔFVC of more than 25% is thought to reflect diaphragmatic weakness [11,25]. Before MRI, a Dwyer pressure gauge was used according to ATS/ERS standards to measure maximum static inspiratory (MIP) and expiratory pressures (MEP) [26]. Results are expressed in kilopascal (kPa). The carbon dioxide (CO2) fraction in the expired gas was measured with a capnograph (ms-capno, Viasys Healthcare, Wurzberg, Germany) at maximum expiration. In the absence of ventilation irregularities, the expiratory CO2 approximates the arterial CO2 pressure. A daytime expiratory CO2 over 6.0 kPa suggests hypercapnia and chronic alveolar hypoventilation [27].

### MRI and imaging analysis

Scanning was performed with a 3T GE Signa 750 MRI (General Electric Healthcare, Milwaukee, USA) using the whole-body coil for radio-frequency excitation and a 32-channel torso coil for signal reception. First, a 3-plane localizer was performed during a maximum inspiratory movement (i.e. a five-second breath-hold scan); all subsequent volumes imaged were based on this localizer. Second, shimming was performed on this localizer, and shim settings were maintained throughout scanning. To evaluate changes in lung shape and volume, two static scans were acquired. These use two 12-second breath-hold scans covering the entire thoracic region acquired at end-inspiration and end-expiration in a 3D RF-spoiled gradient echo sequence with TR/TE = 1/0.5 ms, flip angle 2°, sagittal volume acquisition with 3 mm slice thickness, 1.5 mm slice separation between slices and planar pixel resolution between 1.4x1.4 and 1.5x1.5 mm^2^. Overall acquisition time per patient was 20 minutes.

Each lung was segmented manually at inspiration and expiration using 3D Slicer (http://www.slicer.org), with segmentation being performed every second slice in the axial plane [28]. A full 3D segmentation was reconstructed by interpolating the individual segmentation slices in the cranial-caudal axis. Using the 3D lung segmentations, the length and volume of each independent lung was estimated along the main axes of the MRI acquisition (cranial-caudal, anterior-posterior and left-right). To cope for variations in lung size due to inter-subject anatomical variations, each length at maximum inspiration was divided by the corresponding length at maximum expiration. Therefore the normalised value is a rate of length increase compared with the expiration point (e.g. a ratio of 1.2 would mean an increase of 20% in length). Because of the assumption that the chest-wall is responsible for changes in volume in the anterior-posterior and left-right directions, and the diaphragm expands the lung in the cranial-caudal directions, it is possible to study the contributions to volume changes of the chest-wall and the diaphragm individually.

### Statistical analysis

Data were analyzed using SPSS version 21 (SPSS, Chicago, IL, USA) and are presented as medians with ranges, or as numbers with percentages. The Mann-Whitney test was used to analyze differences in PFT results and MRI findings between patients and volunteers. The Spearman’s correlation coefficient (r) was used to calculate the relationship between PFT outcomes and MRI results in Pompe patients. A p-value <0.05 was considered statistically significant.

## Results

### Study population

Table 1 shows the characteristics of the Pompe patients and the volunteers. All Pompe patients had an acid alpha-glucosidase deficiency and all patients carried the common mutation c.-32-13T > G in one GAA allele and a second pathogenic mutation in the second allele. In five patients this second pathogenic mutation was c.525delT, in two patients c.1548G > A, and the other three patients carried a different second mutation. None of the patients was currently smoking and two patients had smoked in the past. None of the patients or volunteers had co-morbidities that could influence the function of the diaphragm.

**Table 1:** **Patient characteristics and PFT results in patients and volunteers**

	**Patients**	**Volunteers**	**P**-**value**
Age (years)	46 (32-66)	43 (27-55)	0.25
Gender, (% males)	5 (50)	3 (50)	1.0
Height (cm)	178 (154-196)	177 (175-190)	0.39
Weight (kg)	73 (61-88)	85 (65-94)	0.13
BMI (kg/m^2^)	23.4 (20.6-25.4)	24.9 (21-25.7)	0.18
Duration of the disease (years)	16 (9-30)	-	-
Duration of ERT (years)	5.5 (0-7)	-	-
Wheelchair-dependent (%)	1 (10)	0 (0)	0.79
Ventilator-dependent (%)	3 (30)	0 (0)	0.37
*Pulmonary function test*			
FVC_sitting_ (%)	60 (45-84)	102 (92-111)	0.001
FVC_supine_ (%)	43 (27-70)	102 (87-113)	0.001
ΔFVC (%)^a^	33 (11-44)	0 (0-10)	0.001
FEV_1__sitting_ (l/s)	59 (42-80)	98 (85-117)	0.001
FEV_1__supine_ (l/s)	40 (30-63)	91 (76-112)	0.001
MIP (kPa)	6.9 (3.9-8.3)	8.6 (6.4-11.8)	0.07
MEP (kPa)	10.0 (6.4-11.8)	12.5 (10.3-14.2)	0.02

Continuous variables are expressed as median and range, categorical variables as number and percentage. BMI = body mass index, ERT = enzyme replacement therapy, FVC = forced vital capacity, FEV_1_ = forced expiratory volume in one second, MIP = maximum static inspiratory pressure, MEP = maximum static expiratory pressure.

^a^ΔFVC is calculated as (FVC_sitting_-FVC_supine_)/FVC_sitting_ x 100%.

### PFT

Table 1 shows PFT in sitting and supine positions. In both these positions, patients had lower median values for FVC and FEV_1_ than healthy volunteers did (p = 0.001). The median ΔFVC was higher in Pompe patients (p = 0.001). The median MEP was lower in patients (10.0 kPa) than in volunteers (12.5 kPa, p = 0.02). The median MIP showed a trend towards a lower median value for the patients (6.9 kPa) relative to the healthy volunteers (8.6 kPa, p = 0.07). Three patients had a expiratory CO2 fraction over 6.0 kPa and two of these patients were ventilator-dependent.

### MRI

**Figure 1:**
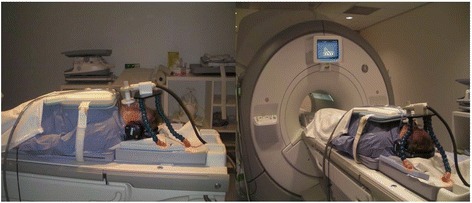
Line-up during the MRI Patients were placed in supine position in the MRI scanner with an MRI-compatible spirometer positioned just above the head.

**Figure 2:**
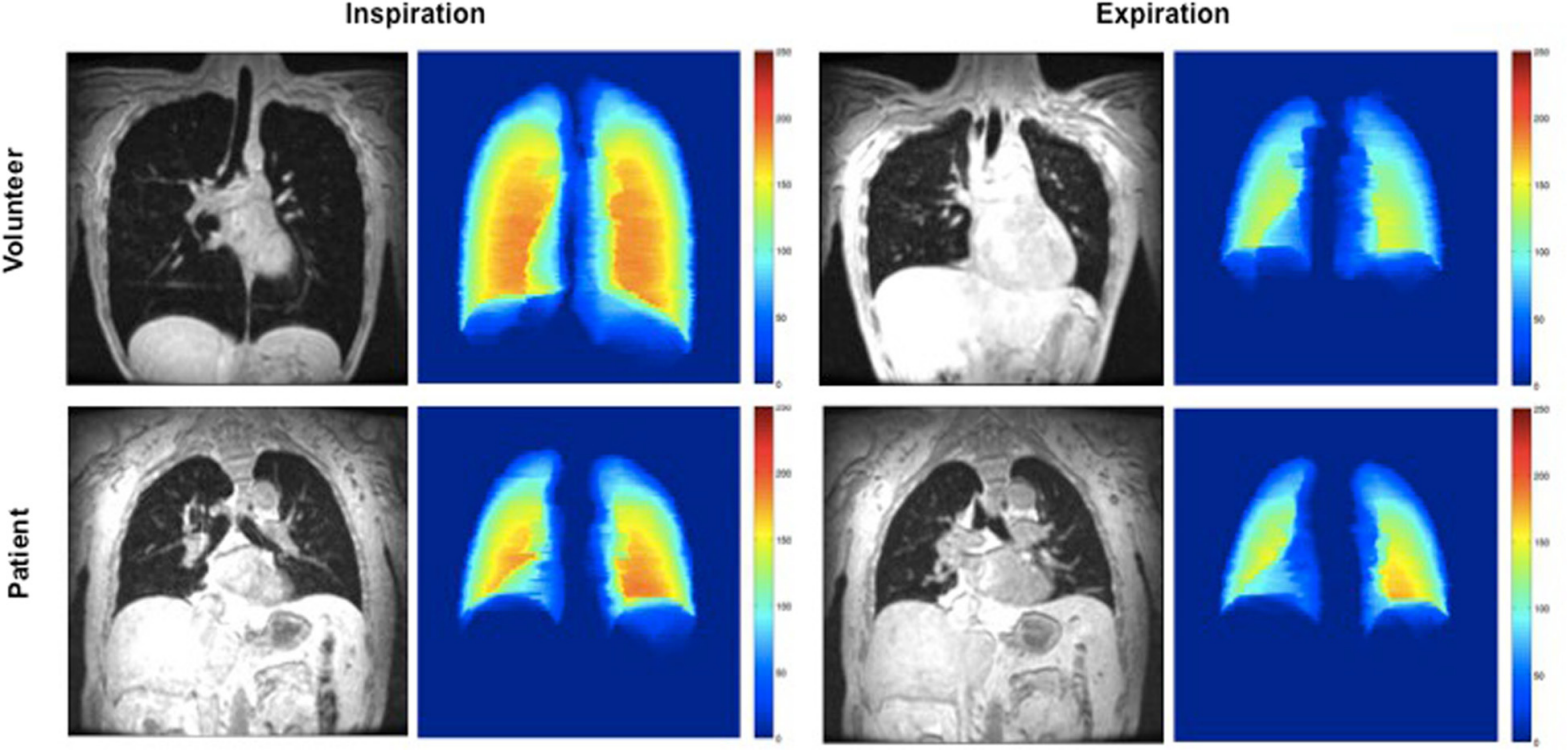
MR images and color maps at maximum inspiration and expiration MR images during 12-second breath-holds in inspiration and expiration in a patient with Pompe disease and a healthy volunteer. The color maps represent the thickness of the segmentation in the anterior-posterior axis (red being the thickest and blue being the thinnest). Note the limited increase in vertical length in the Pompe patient relative to the increase in the healthy volunteer.

Figure 1 shows the line-up during the MRI. Participants were placed in supine position with the MRI-compatible spirometer positioned above them. Figure 2 shows coronal slices through the carina for the breath-hold scans with the corresponding color plots of a Pompe patient and a volunteer. In the Pompe patient there was hardly any displacement of the diaphragm. Additional file 1 in the online data supplement shows the color plots of each individual subject and Additional files 2 and 3 demonstrate two examples of dynamic MRI scans in a healthy volunteer and a patient with Pompe disease.

**Figure 3:**
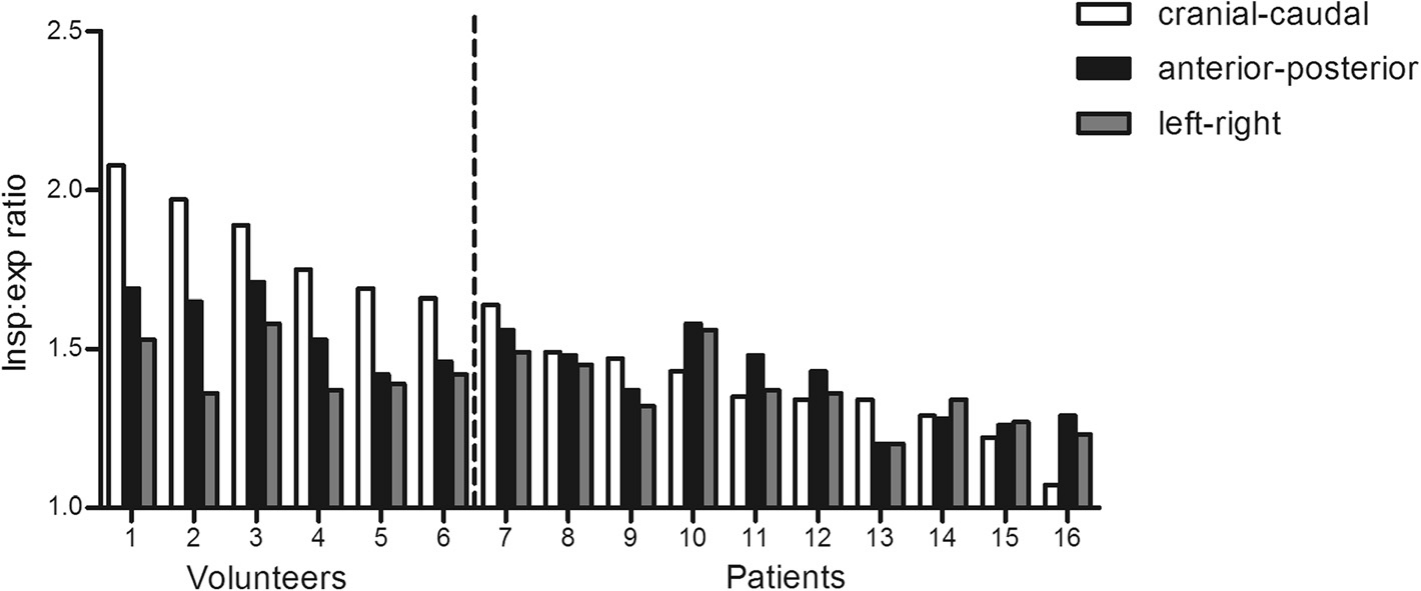
Ratios between inspiration and expiration in three directions for patients and volunteers measured with MRI. The length ratios between inspiration and expiration in the cranial-caudal direction (white bars), anterior-posterior direction (black bars) and left-right direction (grey bars) are shown for individual patients and volunteers. Volunteers are numbered 1 to 6 and patients 7 to 16. The length ratios are calculated by dividing the median length during inspiration by the median length during expiration for each axis.

Figure 3 shows the changes per individual in the three chest-cage directions between inspiration and expiration. In volunteers, the main contributor to the changes in lung volume was the diaphragm (white bars). In most Pompe patients, these changes were due mainly to the thoracic muscles (grey and black bars), but, as Figure 3 shows, these patients had a large variety in diaphragmatic and chest-wall movements. The median cranial-caudal length change, representing diaphragmatic displacement before normalization for lung size, was 82 mm (range 46-90 mm) in volunteers and 28 mm (range 5-49 mm) in patients (p = 0.002). The median anterior-posterior length change was 37 mm in volunteers (range 25-42 mm) and 18 mm (range 13-31 mm) in patients (p = 0.006); the median left-right length change was 24 mm in volunteers (range 21-34 mm) and 17 mm (10-26 mm) in patients (p = 0.02).

**Figure 4:**
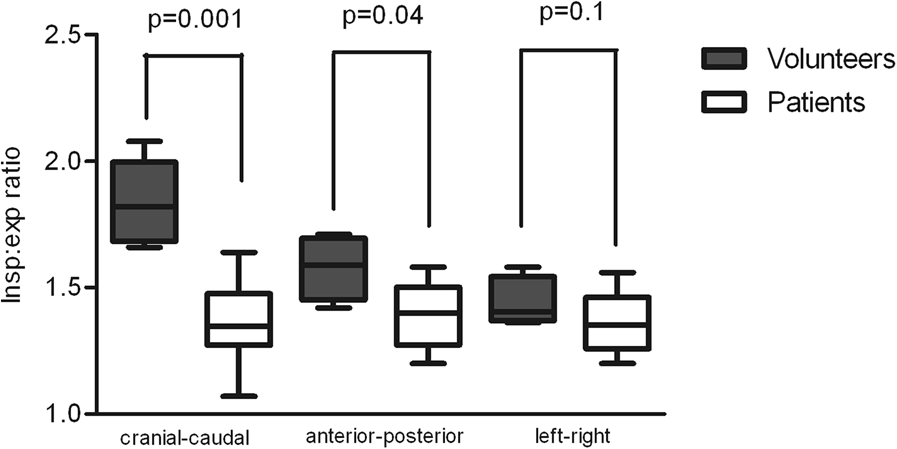
Median ratios between inspiration and expiration in three directions for both groups. This figure shows the same ratios as Figure 2, but now for the groups of Pompe patients and volunteers. The box plots represent the median with the range. The Mann-Whitney test was used to calculate the difference in each direction between patients and volunteers.

Figure 4 shows the different length ratios after normalization for lung size. The cranial-caudal length ratio between inspiration and expiration (representing diaphragmatic displacement) was lower in Pompe patients (median 1.35, range 1.07-1.64) than in volunteers (median 1.82, range 1.66-2.08) (p = 0.001). While the anterior-posterior length ratio was also lower in patients (median 1.40, range 1.20-1.58) than in volunteers (median 1.59, range 1.42-1.71) (p = 0.04), the left-right length ratio did not differ significantly between patients (median 1.35, range 1.20-1.56) and volunteers (median 1.41, range 1.36-1.58) (p = 0.1). In the three Pompe patients who were ventilator-dependent the cranial-caudal length ratio was lower than in the other Pompe patients (median 1.22 versus 1.43, p = 0.02). These ventilator-dependent patients had a longer duration of the disease (median 29 years versus 15 years). There was no correlation between the cranial-caudal length ratio and the duration of ERT.

### Correlation between PFT and MRI

**Figure 5:**
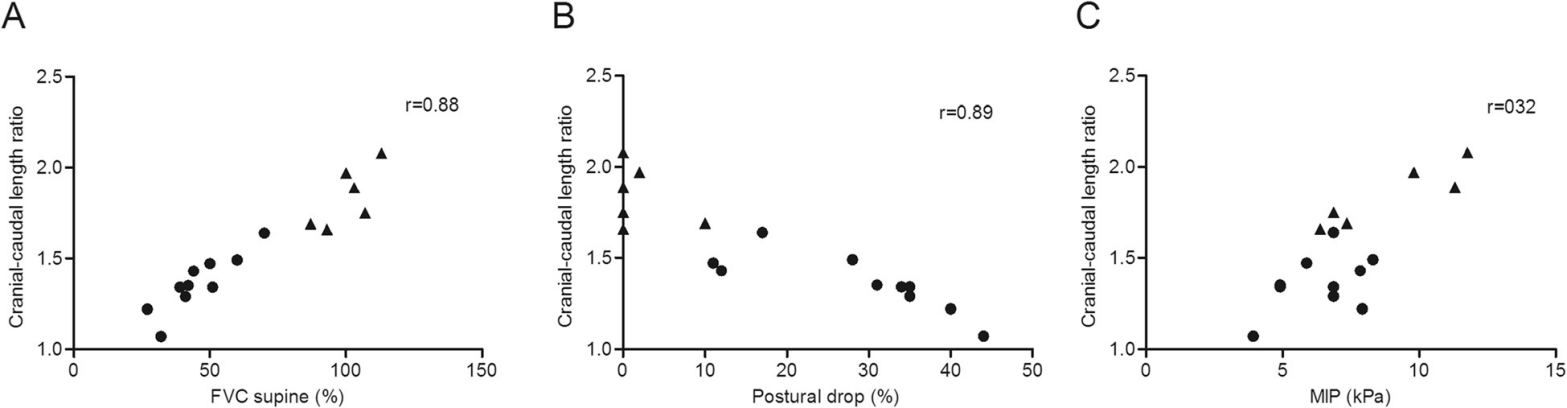
Correlation between cranial-caudal length ratios and FVC supine **(A)**, postural drop **(B)** and MIP **(C)**. The dots represent patients and the triangles volunteers. Spearman’s correlation coefficient (r) was used to calculate the correlation between the cranial-caudal length ratio versus FVC in supine position, the postural drop (ΔFVC) and MIP. As these calculations were performed only in the Pompe patients, the volunteers were excluded for these analyses. FVC = forced vital capacity, MIP = maximum static inspiratory pressure.

As Figure 5 shows, ΔFVC and FVC supine were strongly correlated with the cranial-caudal length ratio (r = 0.89 and r = 0.88, p < 0.001) in Pompe patients, but there were no correlation between MIP and the cranial-caudal length ratio (r = 0.32, p = 0.37), MEP and cranial-caudal length ratio (r = 0.23, p = 0.53), or FVC sitting and cranial-caudal length ratio (r = 0.46, p = 0.18). The only significant correlation regarding the anterior-posterior length ratio was with FVC supine (r = 0.74, p = 0.02).

## Discussion

Our study shows that MRI can be used as an innovative tool to gain greater insight into involvement of the diaphragm in Pompe disease. It was demonstrated that the diaphragmatic function is severely impaired and in some patients there was even hardly any displacement of the diaphragm. To a lesser extent, movement of the anterior chest-wall was reduced. Our results suggest that diaphragmatic displacement measured with MRI is strongly correlated with the postural drop and FVC in supine position measured with common spirometry.

Decreased pulmonary function is an important feature of Pompe disease. Ten or 15 years after onset, half of the adult patients with Pompe disease require ventilator assistance. The main cause of death in this group of patients is respiratory failure, a process in which dysfunction of the diaphragm is considered to play an important role [8,9,29]. Our MRI study suggests that the function of the diaphragm in Pompe disease is more impaired than that of the thoracic musculature. It is not clear how and why the diaphragm muscles are more severely affected than the other respiratory muscles. Our study supports a recent study describing atrophy of the diaphragm and reduced lung height on static MRI and computed tomography scans in patients with Pompe disease. In this latter study semi-quantitative scoring scales were used, and computed tomography was used to measure lung height in one direction [30]. In our study MRI was performed under spirometry control and lung-shape variations were quantified in three directions. This enabled us to show that the cranial-caudal movement related to diaphragmatic function in patients with Pompe disease is impaired more than the anterior-posterior motions of the anterior chest-wall. Similarly, the correlation we found between ΔFVC and FVC in supine position and our MRI results suggest that both these parameters might be used as an indirect tool for determining diaphragmatic function, with the advantage that MRI also visualizes diaphragmatic and chest-wall movements.

A striking finding in our study was that displacement of the diaphragm was extremely impaired in some of the patients, while still residual pulmonary function in supine position was measurable. This could have important consequences when therapy comes in place and might explain why pulmonary function, particularly in supine position, responds poorly to ERT in some of the Pompe patients. Therefore, more studies are required to investigate at what stage the diaphragm and other respiratory muscles become affected in Pompe disease; especially since it has been shown that response to ERT is better in patients who are less severely affected [3,13,31]. Another intriguing question is how MIP and MEP relate to diaphragmatic weakness. It has been hypothesized that these parameters might be better predictors for diaphragmatic weakness than FVC in supine position [30]. Our study implied a weak correlation between MIP or MEP and diaphragmatic displacement. A possible explanation could be that MIP reflects both the strength of the diaphragm and other inspiratory muscles, while the cranial-caudal length ratio only reflects diaphragmatic displacement. In an earlier study we found a positive correlation between FVC in upright position and MIP an MEP [3]. Larger studies are required to explore this relationship in more depth. Comparison of diaphragmatic involvement in patients with Pompe disease to those with other neuromuscular disorders such as Duchenne Muscular Dystrophy might provide insight whether onset and the extend of diaphragmatic involvement is disease specific.

A limitation of our pilot study is that we selected a relatively small number of adult Pompe patients with variable degrees of respiratory dysfunction (FVC in supine position: 27 to 70% of normal). This subset of patients may not be fully representative for the total group of Pompe patients. In subsequent studies also patients with normal or close to normal respiratory function need to be studied to get more insight at what stage of the disease the diaphragm becomes affected. The use of MRI to evaluate diaphragmatic and chest-wall movements has some limitations. Contraindications such as metal implants, invasive ventilation and claustrophobia make it impossible to scan certain patients. Moreover, patients need to be able to perform spirometry in supine position. In next studies it might also be considered to include other techniques to measure lung and respiratory muscle function in addition to spirometry such as sniff nasal inspiratory pressures, transdiaphragmatic pressures or transdiaphragmatic twitch pressures [32]. Prigent et al. showed that transdiaphragmatic pressures and transdiaphragmatic twitch pressures correlated well with all spirometry volumes and non-invasive maximal pressures in adult patients with Pompe disease [33]. Whether transdiaphragmatic pressure measurements show a better correlation with the cranial-caudal length ratio measured with lung MRI than with spirometry data needs further investigation.

## Conclusions

MRI appears to be an innovative tool to visualize diaphragmatic dynamics in Pompe patients and to study chest-wall and diaphragmatic movements in more detail. Our data indicate that diaphragmatic displacement can be very severely impaired in patients with Pompe disease and might explain why FVC responds poorly to ERT in some of the patients. As MRI adds detailed dynamic and structural information to data obtained by pulmonary function tests, particularly of the diaphragm, it may serve as a valuable tool in providing new insights in when the diaphragm starts to be involved in the disease process and on its responsiveness to therapy. It may also serve as a prognostic tool. More research is warranted to explore these topics.

## Additional files

Additional file 1:
**Color maps made at maximum inspiration and expiration.**
Additional file 2:
**Dynamic MRI in a Pompe patient during an FEV1 measurement.**
Additional file 3:
**Dynamic MRI in a healthy volunteer during an FEV1 measurement.**

## Abbreviations

ΔFVC: Postural drop in forced vital capacity from sitting to supine position
BMI: Body mass index
ERT: Enzyme replacement therapy
FVC: Forced vital capacity
FEV_1_: Forced expiratory volume in one second
MIP: Maximum static inspiratory pressure
MEP: Maximum static expiratory pressure
MRI: Magnetic resonance imaging
PFT: Pulmonary function test
PFT: Pulmonary function test
