# Supplementary material for: Lung MRI and impairment of diaphragmatic function in Pompe disease
Source: BMC Pulm Med. 2015 May 6;15:54. doi: 10.1186/s12890-015-0058-3 (PMC4428089; doi:10.1186/s12890-015-0058-3)

Volunteers

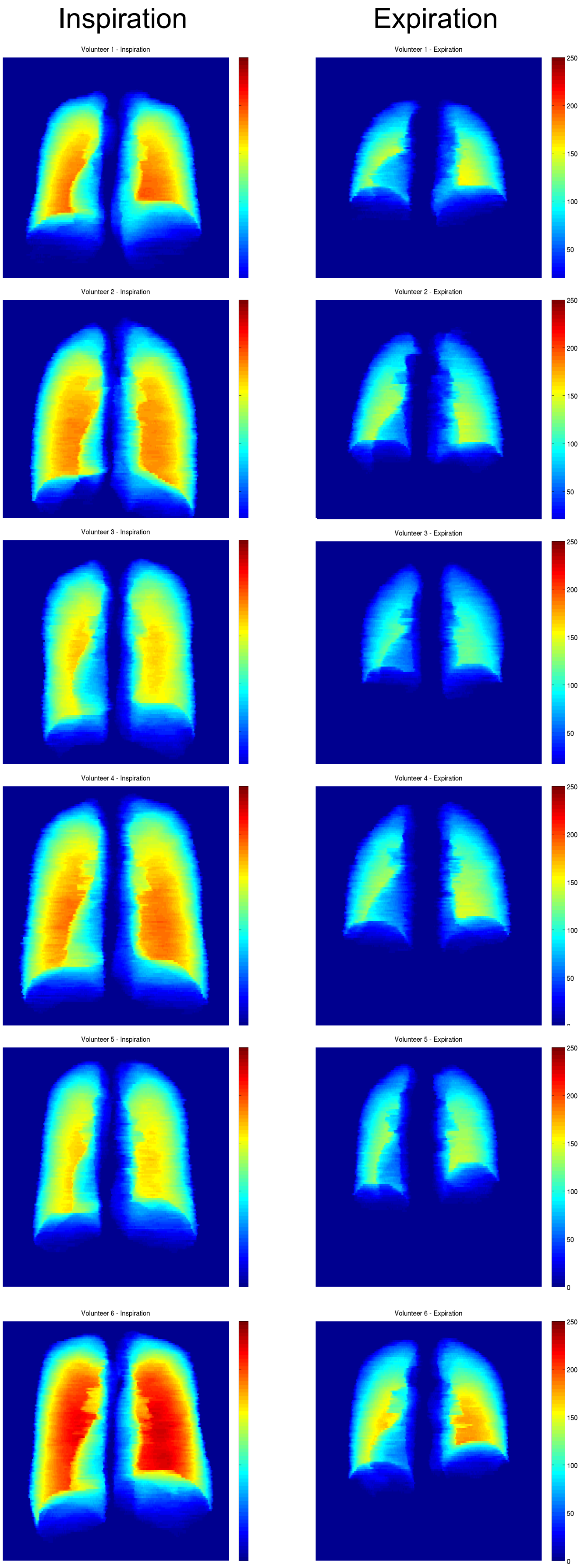

Inspiration
Expiration
Volunteer 1 - Inspiration
Volunteer 1 - Expiration
Volunteer 2 - Inspiration
Volunteer 2 - Expiration
Volunteer 3 - Inspiration
Volunteer 3 - Expiration
Volunteer 4 - Inspiration
Volunteer 4 - Expiration
Volunteer 5 - Inspiration
Volunteer 5 - Expiration
Volunteer 6 - Inspiration
Volunteer 6 - Expiration
250
200
150
100
50
0

Pompe patients

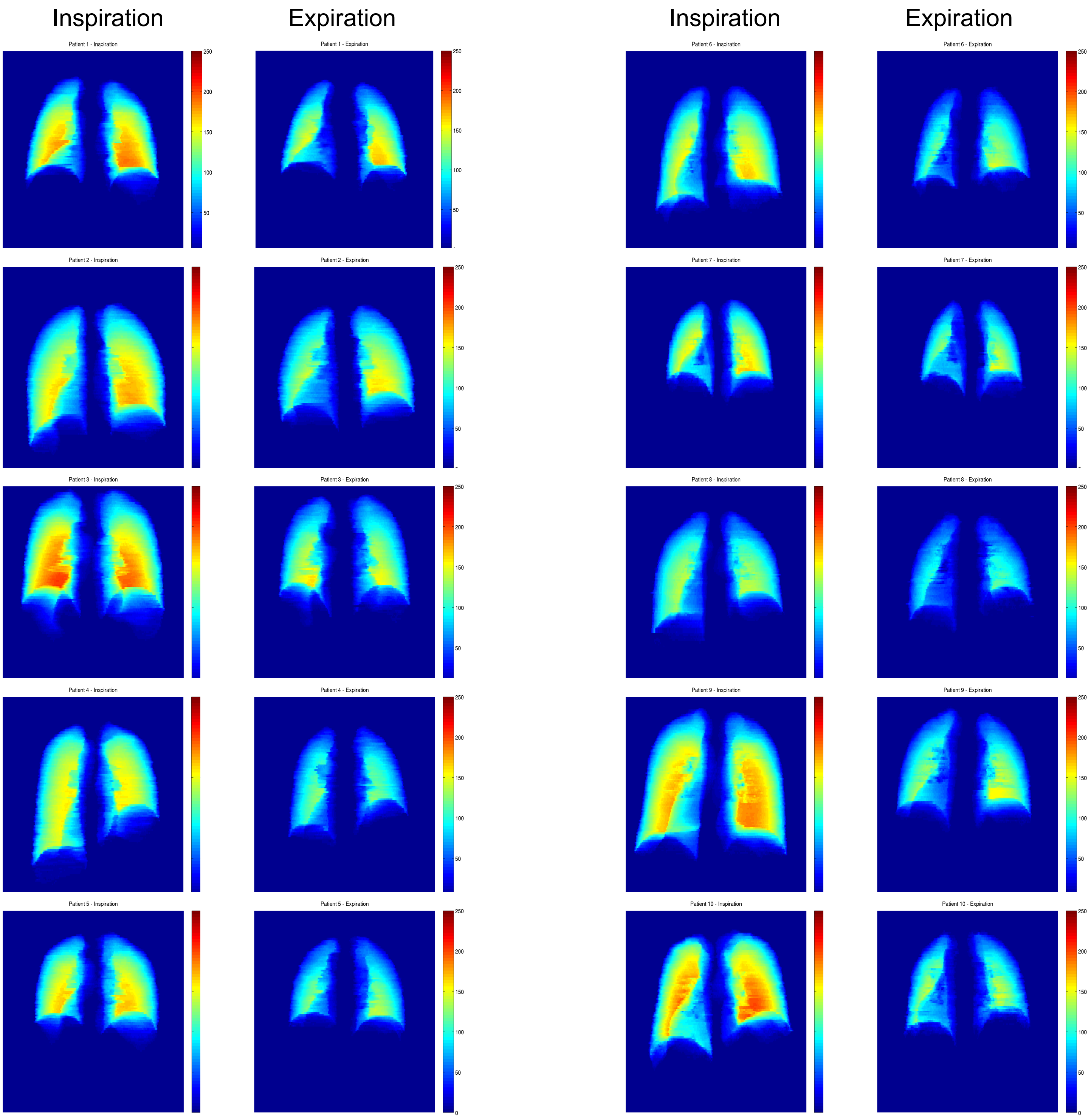

Supplement: Additional file 1: — Color maps made at maximum inspiration and expiration. [file 12890_2015_58_MOESM1_ESM.pdf]
